# Expression in eukaryotic cells and purification of synthetic gene encoding enterocin P: a bacteriocin with broad antimicrobial spectrum

**DOI:** 10.1186/s13568-018-0729-6

**Published:** 2019-01-07

**Authors:** Abbas Tanhaeian, Mohammad Sadegh Damavandi, Davood Mansury, Kiarash Ghaznini

**Affiliations:** 1 Department of Biotechnology and Plant Breeding, Faculty of Agriculture, Ferdowsi University of Mashhad, Mashhad, Iran; 2 Department of Microbiology, School of Medicine, Isfahan University of Medical Sciences, Isfahan, Iran; 3 Department of Medical Laboratory Sciences, Varastegan Institute for Medical Sciences, Mashhad, Iran; 4 Antimicrobial Resistance Research Center, Mashhad University of Medical Sciences, Mashhad, Iran

**Keywords:** Bacteriocin, Antimicrobial peptides, CHO cell, Expression, Enterocin P

## Abstract

Due to the emergence of multidrug-resistant bacteria, treatment options for infectious diseases are decreasing. Bacteriocins are small antimicrobial peptides produced by numerous bacteria that offer alternative therapeutic strategies to combat multidrug-resistant bacterial infections. We evaluated the cloning, functional expression, and antimicrobial activities of enterocin P (EntP), a class II bacteriocin member, in Chinese hamster ovary (CHO) cells. A synthetic gene matching CHO cell codon usage was designed from the known mature amino acid sequence of EntP and cloned into the protein expression vector pcDNA™3.1(+). CHO cells were transformed with the recombinant plasmid and cultured, and the recombinant protein was purified by affinity chromatography. Antimicrobial activities of the recombinant EntP were evaluated on Gram-positive, Gram-negative, and multidrug-resistant pathogens. Recombinant EntP inhibited growth of a variety of bacteria, including pathogenic species known to cause nosocomial infections, often with multidrug-resistant strains. In addition, recombinant EntP demonstrated broad antimicrobial activities in both high salt medium and human plasma and was stable at high temperatures. The broad antimicrobial activity and stability of EntP make it an attractive therapeutic candidate, particularly for treatment of multidrug-resistant bacterial infections.

## Introduction

Multidrug-resistant pathogenic bacteria are a major threat to global health and are rising to dangerously high levels worldwide (Jasovsky et al. 2016). The World Health Organization 2017 fact sheet warns that new antimicrobial resistance mechanisms are emerging and spreading globally, threatening our ability to treat common infectious diseases. Several pathogenic bacteria have developed resistance to multiple antibiotics. In addition, a lack of new antimicrobial agents will inevitably result in increased resistance to existing antibiotics (White et al. 2011). The use of broad-spectrum antibiotics can damage the human microbiota, which is critical in host health. Significant evidence demonstrates that administration of broad-spectrum antibiotics may lead to impaired microbiota and compositional and functional disturbances. The potential link between the use of broad-spectrum antibiotics and increasing incidence of atopic and autoimmune diseases is a specific reason for concern (Krishnamoorthy et al. 2018; Willing et al. 2011). Consequently, new antimicrobials are needed. An option that can no longer be ignored is a subgroup of antimicrobial peptides (AMPs) known as bacteriocins.

Bacteriocins are a heterogeneous group of ribosomally-synthesized AMPs or complex proteins secreted by bacteria that exhibit antimicrobial activity against similar or closely-related species through numerous mechanisms (Micenkova et al. 2014; Shabir et al. 2018). Bacteriocins produced by Gram-positive bacteria are typically classified into three classes: The Class I bacteriocins, also known as lantibiotics, include lanthionine, methyllanthionine, dehydroalanine, and 2-aminoisobutyric acid; the Class II bacteriocins are small non-lanthionine-containing proteins and are divided into five subclasses (IIa/b/c/d/e); and the Class III bacteriocins are large heat-labile proteins, often with enzymatic activity (Cotter et al. 2013). Bacteriocins have net positive charges, which makes them useful for various applications. Bacteriocins have been shown to be active against human and animal bacterial pathogens, either of the same species (narrow spectrum), or other species (broad spectrum). Bacteriocins are highly selective, are effective against multidrug-resistant pathogens, and are non-toxic, which makes them important lead compounds for drug development (Bowdish et al. 2005; Mathur et al. 2018).

Bacteriocins produced by *Enterococcus* spp. are termed enterocins. Most enterocins are class II type. They are generally stable over a wide range of temperatures, pHs, and following lyophilization. These compounds have selective antimicrobial activity, and use this feature to attack *Clostridium* (*C.*) *perfringens*, *Staphylococcus* (*S.*) *aureus,* and especially species of the food-borne pathogenic bacteria including *Clostridium* (*C.*) *botulinum* and *Listeria* (*L.*) *monocytogenes* (Franz et al. 2007; Hu et al. 2010). Enterocins are usually cationic amphiphilic molecules that kill bacterial cells by inserting and accumulating in the membrane, causing increased permeability and loss of barrier functions (Montalban-Lopez et al. 2012).

Short, non-toxic, and highly effective AMPs are needed to overcome antibiotic resistance issues. Here we investigated the expression, purification, and antibacterial effects enterocin P (EntP), a class II cateriocin and used Chinese hamster ovary (CHO) cells as a production platform. CHO cells are widely used for recombinant biopharmaceutical protein production because they are effective protein producers, are suitable for large-scale production, and can be adapted to grow in serum-free and chemically-defined culture media. Also, this mammalian expression system is tolerant to pressure, temperature, pH, and oxygen level changes during manipulation (Kim et al. 2012; Lai et al. 2013).

## Materials and methods

### In-silico analysis tools

The first step for in silico analysis was to search in the Antimicrobial Peptide Database (APD) (http://aps.unmc.edu/AP/main.php) for sequences with high similarities to the target bacteriocin. EntPBLAST sequence homology searches using the NCBI protein BLAST (http://www.ncbi.nlm.nih.gov/BLAST/) were performed to identify the template AMPs. To analyze nucleotide sequences, determine the correct nucleotide sequence from protein, and design restriction enzyme digestion sites for cloning, CLC Genomics Workbench Version 3.6.5 software (Redwood City, CA, USA) was used. Then the I-TASSER simulations (Roy et al. 2010; Yang et al. 2015), were conducted to generate structure predictions for EntP, where homologous templates were excluded from the threading template library. I-TASSER simulations predicted the B-factor profile (BFP), the 3D model, and the estimated global accuracy for EntP.

### Strains and vectors

*Escherichia* (*E.*) *coli* DH5α cells were grown in LB broth (Sigma Chemical Co., St. Louis, MO, USA) at 37 °C with shaking. CHO cells, pcDNA™3.1(+) vector, *Bam*HI and *Hin*dIII restriction enzymes, and kits for plasmid extraction were purchased from Thermo Fisher Scientific (USA).

### Construction of the expression vector and transformation

To increase recombinant EntP expression, a His tag was generated by GenScript’s OptimumGene software (Genscript^®^, USA) and then chemically synthesized by Generay Biotech (Shanghai, China), according to the codon usage of CHO cells. The optimized gene construct encoding a 44-mer EntP peptide plus a 6-His tail was ligated into the multiple cloning site of the plasmid pcDNA3.1(+) with *Bam*HI and *Hin*dIII restriction sites at the 5′ and 3′ ends, respectively (GenScript, USA). Ligations were performed with T4 DNA ligase (Roche Molecular Biochemicals, Mannheim, Germany). After verification of the correct nucleotide sequence, *E. coli* DH5α cells were transformed with the construct using CaCl2 and grown on LB agar contain-ing 100 μg/ml of ampicillin. Colony-PCR was performed to identify colonies containing the recombinant vec-tor. The recombinant vector, named pcDNA3.1(+)-EntP, was extracted using a plasmid extraction kit (Thermo Fisher Scientific, USA) and used to transform CHO cells.

### Cell culture and transfection

CHO cells were maintained in Dulbecco’s Modified Eagle’s Medium (DMEM) supplemented with 8–12 mM l-glutamine with 100 μg/ml penicillin/streptomycin, and 10% heat-inactivated fetal bovine serum (FBS) (Invitrogen, USA), at 37 °C with 5% CO_2_ and a relative humidity of 95%. The culture media was renewed every third day and cultures were passaged every 4–5 days. Viable cells were counted using 0.5% trypan blue (Sigma Aldrich, USA). Approximately 2.5 × 10^5^ CHO cells were seeded into 35 mm wells and cultured in DMEM until 60–80% confluent before transfection. CHO cells were transiently transfected with 10 μg of pcDNA3.1(+)-EntP in serum-free media using Lipofectamine™ 2000 (Invitrogen, USA) according to the manufacturer’s protocol. Seventy-two hours after transfection the medium was replaced with selective medium containing 400 μg ml^−1^ of neomycin (Sigma Aldrich, USA) to kill untransfected cells. The surviving transformed cells were collected and stored at 4 °C.

### Protein production and purification

After establishing the stable recombinant CHO cells and providing an efficient environment for protein production, the supernatants were collected for protein purification by affinity chromatography. Briefly, the supernatant was adjusted to pH 7.0 by the addition of 1 M sodium phosphate buffer, pH 7.0, and filtered through a 0.45 μm membrane filter (Sartorius Stedim, Germany). A HiTrap column was washed with binding buffer containing 20 mM sodium phosphate, pH 7.0, and then the filtered supernatant was loaded onto the column and chromatographed at a flow rate of 3 ml/min. The column was again washed with binding buffer and the bound proteins were eluted with 100 mM sodium citrate buffer, pH 4.6, and collected in Eppendorf microtubes containing neutralization buffer composed of 1 M Tris–HCl, pH 9. The recombinant EntP peptide containing a 6-His tail was purified with Ni–NTA agarose (QIAGEN, USA). The column was washed with distilled water and equilibrated by passing lysis buffer containing 50 mM potassium phosphate, pH 7.8, 400 mM NaCl, 100 m MKCl, 10% glycerol, and 0.5% Triton X-100 through the Ni–NTA agarose. The supernatant was loaded onto the column and chromatographed at a flow rate of 1 ml/min. The column was washed with lysis buffer containing 10 mM and then 30 mM imidazole, and the proteins were eluted with lysis buffer containing 500 mM imidazole and collected in individual microtubes. Finally, a Vivaspin 20 ultrafiltration spin column (Sartorius Stedim, Germany) was used to concentrate and desalt the eluted protein fractions. The EntP peptide concentration was determined by the Bradford method (Bio-Rad Protein Assay) according to the manufacturer’s instructions, using BSA (Protein Assay Standard II) as the standard.

### SDS–PAGE and Western blot analysis

The protein samples were electrophoresed by 15% SDS–PAGE under reducing and non-reducing conditions and the gels were stained with Coomassie Brilliant Blue (Merck, Germany). For Western blotting, the proteins were transferred onto polyvinylidene fluoride (PVDF) membranes. The membranes were blocked in 2% BSA overnight at 4 °C. To identify the recombinant EntP peptide the membranes were incubated for 60 min at room temperature with horseradish peroxidase (HRP)-labelled antibody (Santa Cruz, USA). Finally, the recombinant peptide was visualized by enhanced chemiluminescence detection system (ECL) method according to the manufacturer’s instructions (Amersham Biosciences).

### Bacterial inhibitory activity of bacteriocin EntP

The minimal inhibitory concentration (MIC) of the purified EntP peptide was evaluated using the broth micro-dilution assay according to the Clinical and Laboratory Standards Institute (Wayne PA, CLSI. 2017). Fourteen bacterial strains were used in the study. Six were clinical isolates including *Salmonella* (*S.*) *typhi, L. monocytogenes, S. paratyphi C, Shigella dysenteriae e, vancomycin*-*resistant enterococci* (*VRE*), and carbapenem-resistant *Pseudomonas* (*P.*) *aeruginosa* (CRSA). The other eight bacterial strains were obtained from the ATCC and include the following: *S. aureus* ATCC-25923, *S. aureus* ATCC29213, *Enterococcus* (*E.*) *faecalis* ATCC29212*, E. coli* ATCC25922, *P. aeruginosa* ATCC27853, methicillin-resistant *S. aureus* ATCC33591, *Acinetobacter* (*A.*) *baumannii* ATCC13304, and *Klebsiella* (*K.*) *pneumonia* ATCC 700603. After 18–24 h of incubation on Mueller–Hinton agar at 37 °C, a single colony from each strain was transferred into Mueller–Hinton broth (MHB) (HiMedia, India) and adjusted to an optical density (OD) of 0.5 McFarland units. The cultures were diluted in fresh MHB to a final concentration of approximately 5 × 10^5^ colony-forming units (CFUs)/ml. Assays were conducted in 96-well microtiter plates at 10 different concentrations of EntP peptide prepared by two-fold dilutions from 0.5 to 512 μg/ml in MHB. Vancomycin and gentamicin were used as positive references to determine the sensitivity of each bacterial species tested. The MIC was determined as the lowest concentration of EntP peptide that inhibited visible growth after overnight incubation at 37 °C. All tests were performed in triplicate.

### Effect of salt, and 50% human plasma on antimicrobial activity

To investigate the activities of the EntP peptide in the presence of high salt concentrations, the MIC was determined as described above, except that fixed concentration of NaCl was added to each well of the microtiter plate. Overnight cultures of *S. aureus* (ATCC 25923) and *E. coli* (ATCC 25922) as Gram-positive and Gram-negative model strains were incubated in MHB with 0, 50, 100, or 150 mM NaCl for 4 h. Bacteria were serially diluted and plated in triplicate on Trypticase soy agar (HiMedia, India) plates. CFUs were counted after 24 h of incubation at 37 °C. The stability of EntP peptide in 50% human plasma was evaluated as previously described (Hou et al. 2011). The human plasma was determined to contain no antimicrobial activity before the test. Then 800 μg/ml of recombinant EntP peptide was diluted 1:1 with fresh human plasma and incubated at 37 °C for 0, 3, or 6 h. After incubation, the antimicrobial activity of each sample was determined by MIC assays with *S. aureus* (ATCC 25923) and *E. coli* (ATCC 25922).

## Results

### In silico prediction and 3D structure design

**Fig. 1:**
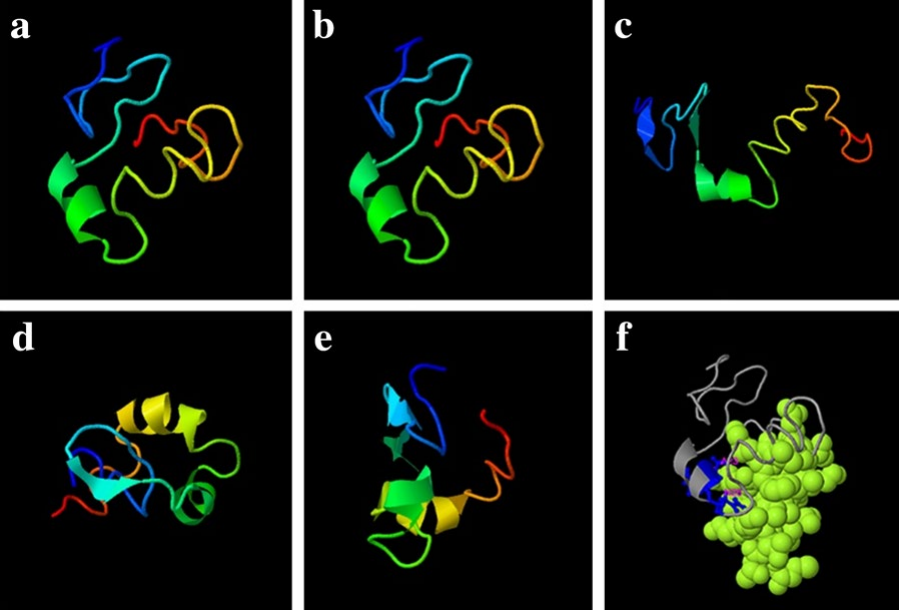
The predicted 3D model and the estimated global accuracy of EntP peptide. **a** Model 1: C-score = − 1.35, **b** model 2: C-score = − 1.86, **c** model 3: C-score = − 2.70, **d** model 4: C-score = − 1.66, **e** model 5: C-score = − 2.93, **f** binding residues are shown in blue ball and stick and the predicted-binding ligand is represented as green-yellow spheres

**Fig. 2:**
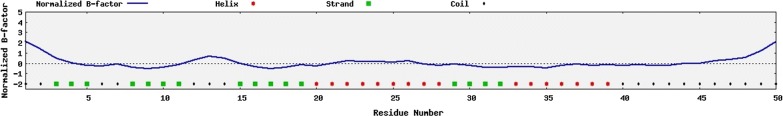
The predicted normalized B-factor profile (BFP); Residue with negative values are relatively more stable than those with positive values in the structure

**Fig. 3:**
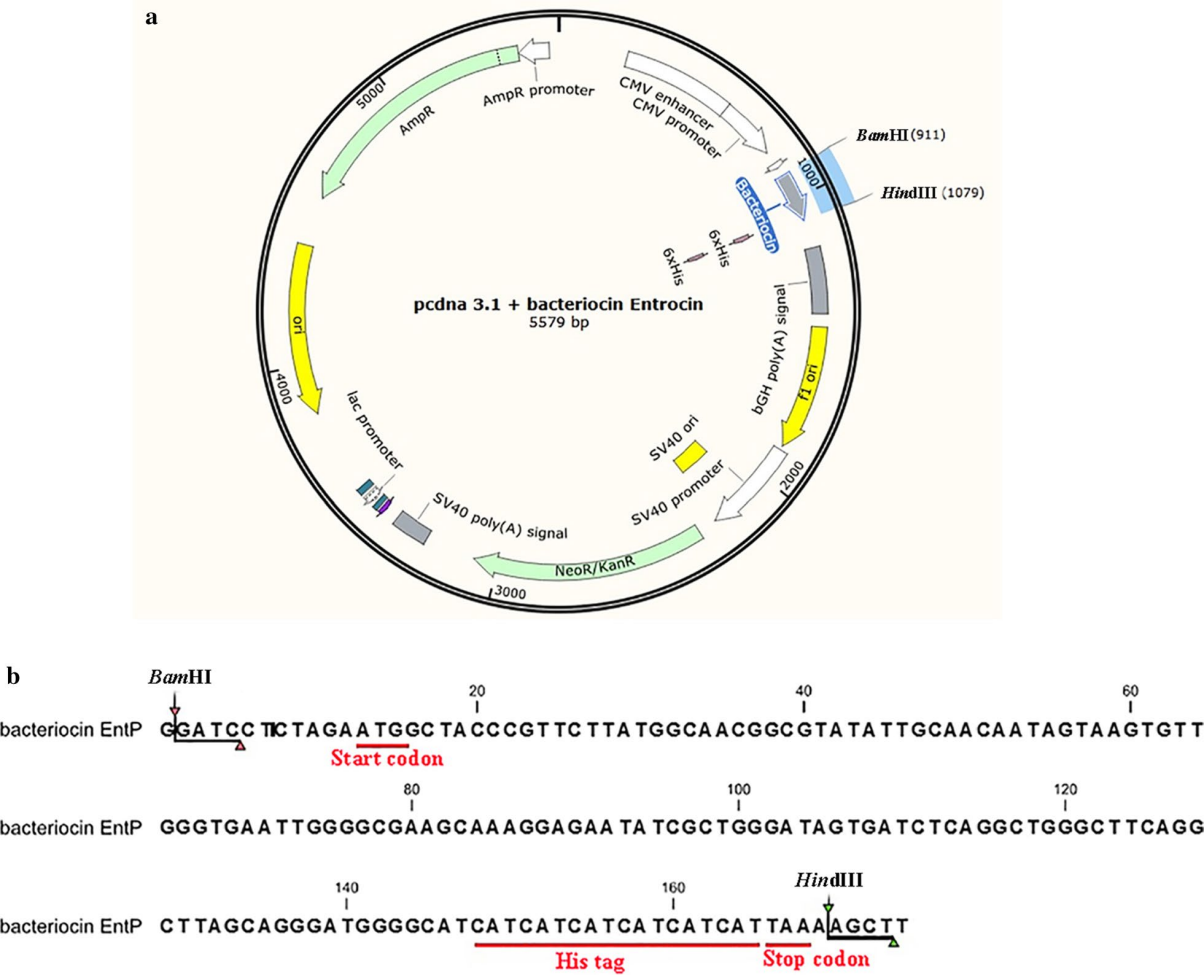
Scheme figure of the pcDNA3.1(+)-EntP plasmid. **a** The EntP coding sequence was ligated into pcDNA3.1(+). **b** The EntP nucleotide sequence with *Bam*H1 and *Hin*dIII restriction sites and C-terminal 6× His tag

The Protein Data Bank (PDB) sequence search using a significant cutoff E-value of 0.01 showed that the deduced amino acid sequence of the putative EntP peptide was approximately 80% similar to curvacin A and 50% similar to carnobacteriocin B2. The top five 3D models predicted by I-TASSER and estimated global accuracy of the models for EntP are shown in Fig. 1a–e. The C-score estimates the accuracy of the I-TASSER predictions. The EntP peptide C-score in (Jack et al. 1996) and C-score > − 1.5 indicates a model of correct global topology. The predicted ligand binding sites are shown in Fig. 1f. The BFP indicates the extent of the inherent thermal mobility of residues/atoms in proteins and was predicted using a combination of both template-based assignment and profile-based prediction. Based on the BFP distributions and predictions, residues with BFP values greater than 0 are less stable in experimental structures than those with BFP values less than 0. The estimated normalized BFP is shown in Fig. 2. The instability index determined the ExPASy Server (Wilkins et al. 1999) was 12.91. This value classifies the protein as stable. The pCDNA-EntP peptide construct is shown in Fig. 3a. The EntP nucleotide sequence containing the 6-His tail the *Bam*HI and *Hin*dIII sites are shown in Fig. 3b.

### Purification and visualization of enterocin P

**Fig. 4:**
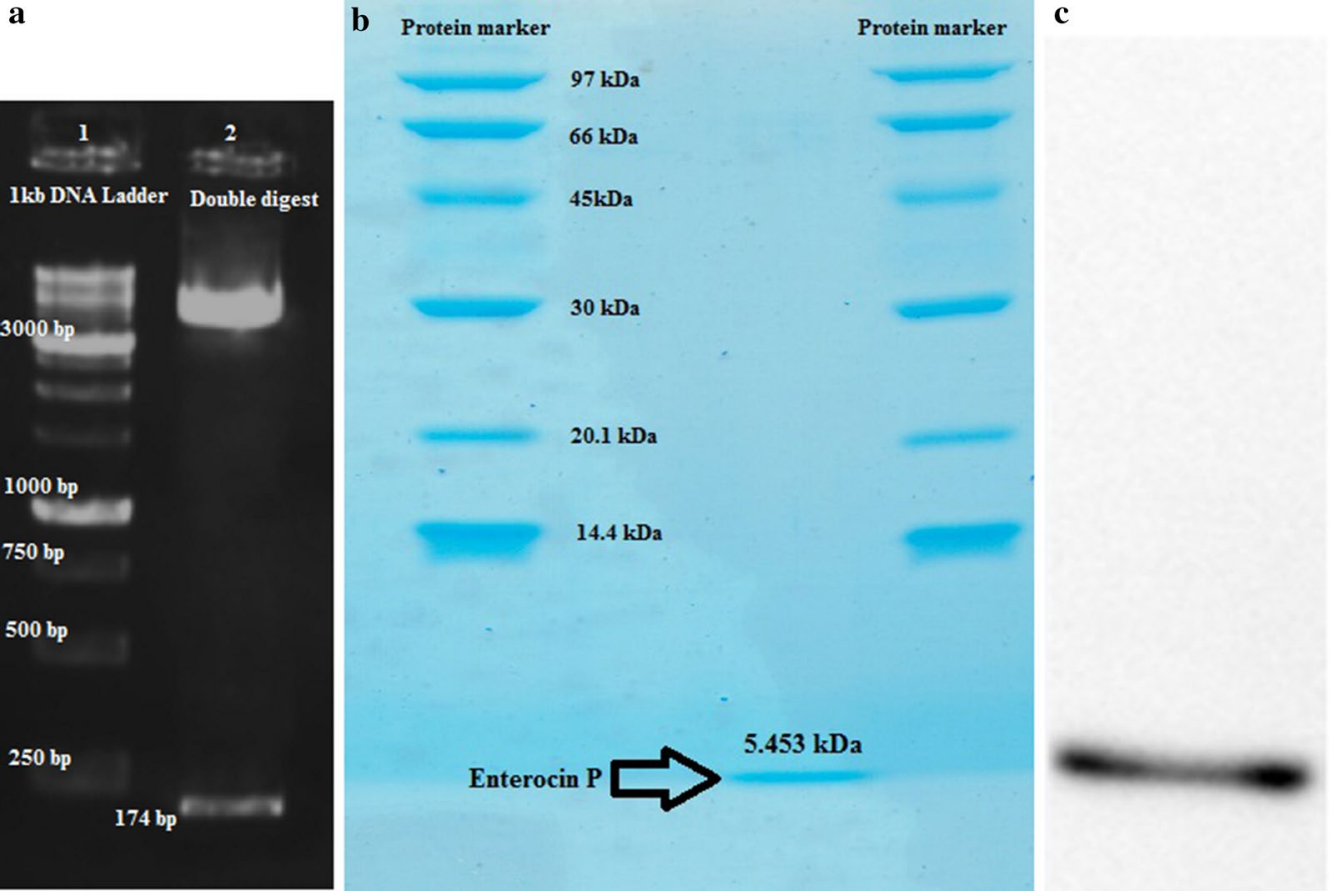
Agarose and SDS-PAGE gels of *Bam*H1 and *Hin*dIII-digested pcDNA3.1(+)-EntP peptide and purified EntP peptide, respectively.** a** The pcDNA3.1(+)-EntP peptide plasmid was digested with *Bam*HI and *Hin*dIII and electrophoresed on an agarose gel; Lane 1: 1 kB DNA ladder; Lane 2: *Bam*H1 and *Hin*dIII-digested plasmid. The 174 bp fragment represents the EntP peptide coding sequence. **b** SDS-PAGE of purified EntP peptide.** c** Western blotting analysis of purified EntP peptide (5.453 KDa)

The pcDNA3.1(+)-EntP plasmid was digested with *Bam*H1 and *Hin*dIII to verify the presence of the EntP coding sequence in pcDNA3.1(+) (Fig. 4a). On SDS-PAGE the purified EntP peptide migrated as a single band with a molecular weight of 5453 Da (Fig. 4b). On Western blots the purified EntP peptide immunoreacted with goat anti-human IgG–HRP (Fig. 4c).

### Antibacterial activity assay

The MICs of purified EntP peptide estimated by a broth micro-dilution assay against important human pathogenic microorganisms are shown in Table 1. All these clinically-isolated and ATCC pathogenic bacteria were inhibited by pure recombinant EntP at MICs ranging from 5.57 to 267.5 μg/ml. Of the fourteen bacterial strains tested, *K. pneumonia* ATCC 700603 and *P. aeruginosa* ATCC 27853 were the most susceptible, followed by *S. aureus* ATCC 25923, while CRSA was the least susceptible.

**Table 1:** Minimum inhibitory concentrations (MICs) of the EntP peptide and various antibiotics

Strain	Antimicrobial activity MIC (ug/ml)					
	EntP	Ampicillin	Ceftazidime	Vancomycin	Piperacillin	Gentamicin
Gram positive bacteria						
*Staphylococcus aureus* ATCC 25923	8	≤ 1	≤ 2	≤ 1	ND	≤ 1
*Staphylococcus aureus* ATCC 29213	8	≤ 1	≤ 2	≤ 1	ND	≤ 1
*Enterococcus faecalis* ATCC 29212	16	≤ 1	≤ 4	≤ 1	ND	≤ 1
*Listeria monocytogenes*	32	2	≤ 4	1	4	8
Gram negative bacteria						
*E. coli* ATCC 25922	128	≤ 2	≤ 2	ND	≤ 8	≤ 1
*Salmonella typhi*	16	2	4	ND	4	≤ 2
*Salmonella paratyphi C*	ND	2	4	ND	≤ 8	≤ 1
*Shigella dysenteriae*	128	4	≤ 2	ND	4	4
*Pseudomonas aeruginosa* ATCC 27853	8	ND	2	ND	≤ 8	≤ 16
Resistant strains						
MRSA ATCC 33591	64	ND	16	≥ 16	ND	≥ 16
*VRE*	64	ND	32	≥ 32	ND	≥ 16
*Pseudomonas aeruginosa VIM*+	256	ND	≥ 32	ND	≥ 128	≥ 16
*Acinetobacter baumannii* ATCC 13304	128	ND	≥ 32	ND	≥ 64	≥ 16
*Klebsiella pneumonia* ATCC 700603	8	> 128	≥ 16	ND	≥ 128	≥ 16

Data are collected as MICs according to the Clinical and Laboratory Standards Institute (CLSI)

*ND* not determined

The antimicrobial activity of EntP peptide in 50% human plasma was not significantly different from that in PBS. Interestingly, the results showed no significant difference activity of in the presence of salt Table 2.

**Table 2:** Effect of salt and plasma on activity of enterocin P

	Activity after treatment with salt and plasma	
	*E. coli*	*S. aureus*
Salt concentration (mM)		
0	+	+
50	+	+
100	+	+
150	−	−
Plasma (h)		
0	+	+
3	+	+
6	+	+

+, presence of activity; −, absence of activity

## Discussion

The increased bacterial resistance to current antimicrobial agents is a major public health problem. For nearly a century antibiotics have been reliable and effective against most bacterial infections. However, after excessive and prolonged use, many bacteria have become resistant to most available antibiotics. In healthcare settings patients are frequently infected with these strains. Since the emergence of this difficult situation, several novel therapeutic options have been explored. AMPs are one alternative to antibiotics to control and fight drug-resistant bacterial infections. Their low cytotoxicity and high antimicrobial and broad-spectrum activities are favorable characteristics for AMPs as potential antibacterial candidates (Chellat et al. 2016; Chiang et al. 2018; Frieri et al. 2017).

In other studies many AMPs, such as enterocin L50 (Basanta et al. 2010), pediocin PA-1 (Beaulieu et al. 2005), and hiracin JM79 (Sanchez et al. 2008) have been expressed in active forms in *Pichia pastoris*, but their production was low. Codon optimization is a beneficial strategy to increase the target protein yield during expression. In the present study we optimized codon usage in a CHO cell line. CHO cells are commonly used to produce high levels of recombinant protein (Butler and Spearman 2014). In this study, we evaluated the antimicrobial activities of the bacteriocin EntP peptide and analyzed its antimicrobial effects. We used pcDNA3.1(+) to optimize expression and peptide secretion in the CHO cells.

The purified EntP peptide exhibited antibacterial activity against all the bacteria tested. The ability of EntP peptide to inhibit the growth of a number of food-borne pathogens, including *L. monocytogenes, S. typhi*, and *S. aureus*, suggests that purified EntP peptide may be a useful tool in the food production industry. The activity observed against gram-negative bacteria is unusual and has been reported for only a few bacteriocins produced in lactic acid bacteria (De Kwaadsteniet et al. 2005; Drider et al. 2006).

The thick peptidoglycan layer of Gram-positive bacterial cell walls contains teichoic or lipoteichoic acids (LTA), whereas Gram-negative bacteria have cell walls rich in lipids and carbohydrates, such as lipopolysaccharide (LPS), resulting in highly electronegative cell walls. Both LPS and LTA provide binding sites at outer membrane surfaces that enable cationic AMPs to reach the target cytoplasmic membrane. In general, AMPs are initially attracted to the bacterial surface by electrostatic interactions between the cationic peptide and the negatively-charged bacterial surface. Addition of histidine to EntP peptide increased its positive charge; therefore, the histidine tail has no negative effect on the peptide’s antibacterial activity (Jamasbi et al. 2014; Park et al. 2011). Cationic peptides are likely attracted to negatively-charged LPS molecules in Gram-negative bacteria and the peptidoglycan layer of Gram-positive bacteria. In this study, details of the peptide structure were further investigated using the I-TASSER protein structure prediction server. The 3D peptide structure analysis in our study showed that the predicted structure of model one with a c-score of − 1.35 has more functional properties than the other models and is probably the form most suitable for binding to bacterial cell walls. A major limitations with AMPs for systemic applications is their possible inactivation by serum or physiological salt concentrations (Yeung et al. 2011). We found that EntP retained its bactericidal activity in the presence of 50% human plasma. Furthermore, EntP retained its antibacterial activity in up to 150 mM NaCl, in contrast to other studies that found the antibacterial activities of gramicidins, human defensin-1, magainins, and bactenecins substantially reduced under similar conditions (Chu et al. 2013). Similar to our results, myxinidin exerted antimicrobial effects against a broad range of bacteria, even under high-salt conditions (Cantisani et al. 2014). Turner et al. reported that addition of 100 mM NaCl resulted in 12- and 100-fold increases in the MICs of LL-37 and human neutrophil peptide-1, respectively, when tested on MRSA (Turner et al. 1998). Also similar to our results, other studies found that native EntA was stable at 100 °C (Rehaiem et al. 2011) and the bactericidal activities of pediocin PA-1 and piscicolin 126 were maintained at higher temperatures (Jack et al. 1996; Kaur et al. 2004). The ability to resist the effects of human plasma, salt, and temperature provide a selective advantage for our peptides for potential therapeutics in physiological conditions.

In conclusion, the characteristics of EntP peptide described in our study demonstrate that it may be a promising candidate for clinical use. The peptide was stable in high salt and heat, which may be beneficial in recombinant expression and purification. The antimicrobial activity of bacteriocin EntP likely correlated with its ability to bind and permeabilize bacterial membranes. We have demonstrated activity against a wide variety of bacteria, including pathogenic species known to account for a large number nosocomial infections, often with multidrug-resistant activities, suggesting broad therapeutic applications.

## Abbreviations

EntP: enterocin P
CHO cells: Chinese hamster ovary cells
AMPs: antimicrobial peptides
APD: antimicrobial peptide database
PVDF: polyvinylidene fluoride
